# PTX3: an inflammatory protein modulating ultrastructure and bioenergetics of human endothelial cells

**DOI:** 10.1186/s12979-019-0144-0

**Published:** 2019-02-02

**Authors:** Albino Carrizzo, Claudio Procaccini, Paola Lenzi, Clorinda Fusco, Francesco Villa, Serena Migliarino, Massimiliano De Lucia, Francesco Fornai, Giuseppe Matarese, Annibale A. Puca, Carmine Vecchione

**Affiliations:** 1 Vascular Pathophysiology Unit, IRCCS Neuromed, 86077 Pozzilli, IS Italy; 2 Laboratorio di Immunologia, Istituto di Endocrinologia e Oncologia Sperimentale, Consiglio Nazionale delle Ricerche (IEOS-CNR), Naples, Italy; 3 IRCSS Fondazione Santa Lucia, Rome, Italy; 4 Department of Translational Research and New Technologies in Medicine and Surgery, University of Pisa, Via Roma 55, 56126 Pisa, Italy; 5 Dipartimento di Medicina Molecolare e Biotecnologie Mediche, Università degli Studi di Napoli “Federico II”, Naples, Italy; 6 Cardiovascular Research Unit, IRCCS MultiMedica, 20099 Sesto San Giovanni, MI Italy; 7 Department of Clinical and Molecular Medicine, School of Medicine and Psychology, Sapienza University of Rome, Rome, Italy; 8 Department of Medicine and Surgery, University of Salerno, Via S. Allende, 84081 Baronissi, SA Italy

**Keywords:** Pentraxin 3, Endothelial cells, Mitochondria, Bioenergetics

## Abstract

**Background:**

Pentraxin 3 (PTX3), an acute-phase inflammation protein produced by several cell types, has long been described as a possible biomarker for age-related cardiovascular and cerebrovascular diseases. Although several mechanisms of action have been identified to date in the vascular and immune systems, the direct effects of PTX3 on isolated endothelial cells at morphological and metabolic levels remain unknown.

**Findings:**

PTX3 induced cytoplasmic vacuolization and dilution of mitochondrial matrix in isolated, human endothelial cells. Moreover, metabolic assays revealed that PTX3 increases respiratory capacity in support of mitochondrial function, and partially sustains the glycolytic pathway.

**Conclusions:**

PTX3 has, per se*,* a direct action on ultrastructural and bioenergetic parameters of isolated endothelial cells. This finding can be associated with our previous demonstration of a deleterious effect of PTX3 on the endothelial layer. More studies are needed to clearly demonstrate any direct correlation between these ultrastructural and bioenergetic changes with endothelial dysfunction, especially with regard to age-related cerebro- and cardio-vascular diseases.

## Introduction

In the last decade, several inflammatory mediators have been implicated in the pathogenesis of age-related cerebro- and cardio-vascular disorders [1–3]. Moreover, the growing body of studies linking inflammation to endothelial activation and loss of nitric oxide bioactivity has promoted investigators to seek evidence on the possible correlation of acute-phase proteins with endothelial-specific alterations [4, 5]. On this point, an elevated level of circulating Pentraxin 3 (PTX3) – a member of a protein superfamily involved in the innate immune response – has been described as a marker of poor prognosis in patients with stable coronary artery disease or heart failure [6–8]. Other studies have reported that PTX3 levels are higher in women with preeclampsia, speculating on its contribution to endothelial dysfunction [9]. Recently, we have reported that PTX3 is directly implicated in the pathogenesis of vascular endothelial dysfunction, through a P-selectin/matrix metalloproteinase-1 pathway [10], demonstrating that the exposure of mouse mesenteric arteries to PTX3 leads to alterations of vascular ultrastructural and impairment of nitric oxide production. Moreover, circulating levels of PTX3 were found to be increased in hypertensive patients, leading us to candidate PTX3 as a novel prognostic marker for arterial hypertension [10]. These findings clearly suggest that the endothelium represents one of the main targets of PTX3 at the vascular level.

However, although it has been demonstrated that ultrastructural alteration of membrane and organelles and modification of bioenergetic parameters represent important determinants of endothelial cell malfunction, no study has focused on the possible modulation of these parameters by PTX3 in human endothelial cells (ECs).

Here, we demonstrate for the first time that PTX3 induces cytoplasmic vacuolization and dilution of mitochondrial matrix. This finding, in association with modification of bioenergetics, might reflect a clear state of EC suffering.

## Material and methods

### Cell culture

Human umbilical vein endothelial cells (HUVECs; Lonza) were cultured in EBM-2 medium (Lonza) at 37 °C in 5% CO_2_/95% air by standard methodologies in 25-cm^2^ tissue culture flasks (50 ml capacity) (Falcon, Becton Dickinson Labware) in the presence of endothelial cell growth supplement [11]. HUVECs, used for experiments after 3–4 passages, were grown to 50–60% confluence before exposure to PTX3 (20 ng/mL for 1 h or 12 h).

### Transmission Electron microscopy (TEM)

HUVEC pellets were fixed in 2% paraformaldehyde and 0.1 glutaraldehyde in 0.1 M PBS, pH 7.4 for 90 min; after washing in PBS, cells were post-fixed in 1% OsO_4_ for 1 h at 4 °C. Then, cells were dehydrated in ethanol and embedded in Epon–araldite. Ultrathin sections were stained with uranyl acetate and lead citrate and examined under a Jeol Jem 100SX transmission electron microscope (Jeol, Tokyo, Japan).

### Mitochondrial bioenergetics and metabolic assays

The bioenergetic profile was measured in HUVEC cells treated or not with PTX3 (20 ng/mL) for 1 or 12 h. Real-time measurements of oxygen consumption rate (OCR) and extracellular acidification rate (ECAR) were made using an XFe-96 Extracellular Flux Analyzer (Seahorse Bioscience). Cells were plated in XFe-96 plates (Seahorse Bioscience) at 20,000 cells/well. OCR was measured in XF medium (non-buffered DMEM medium containing 10 mM glucose, 2 mM L-glutamine, and 1 mM sodium pyruvate) under basal conditions and in response to 5 μM oligomycin, 1.5 μM carbonylcyanide-4- (trifluoromethoxy)-phenylhydrazone (FCCP), or 1 μM antimycin plus rotenone (ant-rot) (all from Sigma-Aldrich). ECAR was measured in XF medium (according to the manufacturer’s instructions) under the basal condition and in response to 10 mM glucose, 5 μM oligomycin, or 100 mM 2-deoxy-D-glucose (2-DG). The OCR profiles were used to determine basal OCR (calculated as the difference between baseline measurements and ant/rot-induced OCR), ATP-linked OCR (calculated as the difference between basal OCR and oligomycin-induced OCR), and maximal OCR (calculated as the difference between FCCP-induced OCR and ant/rot-induced OCR). The ECAR profiles were used to determine basal glycolysis (in the presence of glucose), maximal glycolysis (after the addition of oligomycin), and glycolytic capacity (calculated as the difference between oligomycin-induced ECAR and 2-DG-induced ECAR).

### Statistical analysis

Data are expressed as mean ± S.E.M. (*n* = 5 replicates/sample). Statistical differences were evaluated using the Wilcoxon matched-pairs test.

## Results

**Fig. 1:**
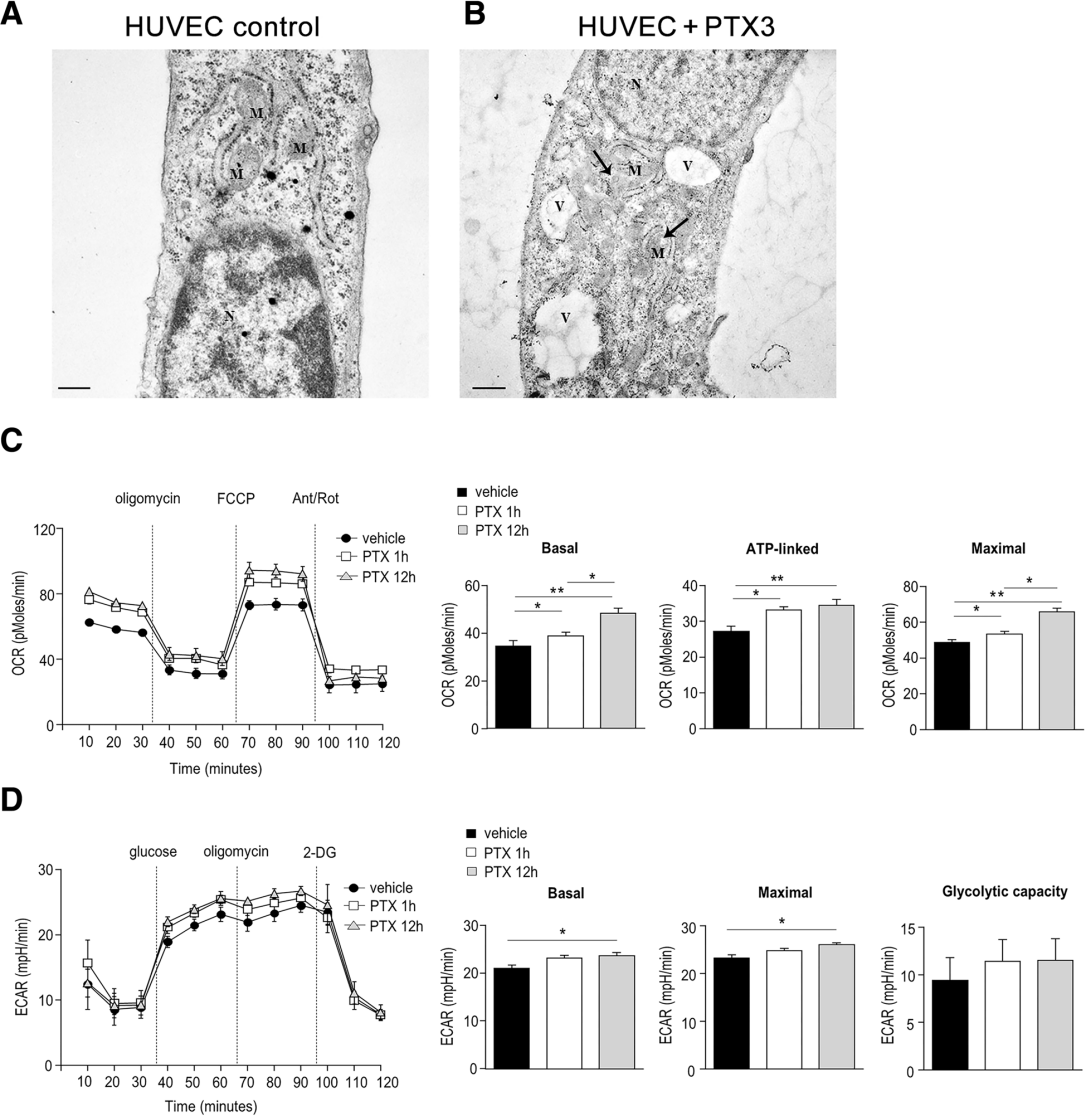
**a, b**) *Effects of PTX3 on HUVEC ultrastructure*. Representative micrographs of control (**a**) and PTX3-treated (**b**) HUVECs. In (**a**), mitochondria are well-conformed, while in a treated cell (**b**) mitochondria appear with diluted matrices (arrows) and the cytoplasm contains large vacuoles. M = Mitochondria, N = Nucleus, V=Vacuole. Scale bar = (**a**) 0.33 μm; (**b**) 0.5 μm. **c**, **d**) *Effects of PTX3 on bioenergetic profile*. In **c**), oxygen consumption rate (OCR) measured in HUVECs exposed or not to PTX3 for 1 or 12 h in real time, under basal conditions and in response to indicated mitochondrial compounds: oligomycin, carbonylcyanide-4- (trifluoromethoxy) -phenylhydrazone (FCCP), or antimycin A plus rotenone (Ant/Rot), using an XFe-96 Extracellular Flux Analyzer. Indices of mitochondrial respiratory function calculated from the bioenergetic profiles (as described in Materials and Methods). In **d**), extracellular acidification rate (ECAR) measured in HUVECs exposed or not to PTX3 for 1 or 12 h in real time under basal conditions and in response to glucose, oligomycin, or 2-deoxy-D-glucose (2-DG). Indices of glycolytic pathway activation calculated from the bioenergetic profiles (as described in Materials and Methods). Data are expressed as mean ± S.E.M. One representative out of two independent experiments. Statistical analysis by Wilcoxon matched-pairs test. (*p<0.05; **p<0.0005)

First, TEM was used to assess the ultrastructural effects of PTX3 on isolated HUVECs. PTX3 induced the development of large cytoplasmic vacuoles: after 12 h of exposure, these were found located particularly close to the mitochondria (Fig. 1a), which clearly had a diluted matrix (Fig. 1b). Since the mitochondrial matrix is a structured, reticular network of proteins that undergoes geometric rearrangement on the basis of metabolic activity and respiratory state [12], we investigated the effect of PTX3 on cellular bioenergetics, measuring OCR – an indicator of oxidative phosphorylation (OxPhos) – and ECAR – an indicator of glycolysis on HUVEC cells exposed to PTX3 for 1 or 12 h. We found that PTX3 significantly enhanced OxPhos, as indicated by increased basal OCR (Fig. 1c, d). After addition of oligomycin, ATP-linked OCR was also increased (Fig. 1c), as was maximal respiratory capacity upon addition of FCCP (Fig. 1d). These findings suggested that PTX3 increases respiratory capacity in order to sustain mitochondrial function under increasing metabolic demand.

We then used the same experimental conditions to evaluate the effects of PTX3 on the activation of glycolysis, the other key metabolic pathway – together with OxPhos – needed to generate cellular ATP. We found that PTX3 induced a tendency to sustain ECAR (Fig. 1e). More in detail, ECAR was slightly increased by PTX3 only after 12 h of exposure, with glycolytic capacity showing an increased, though not statistically significant, trend at both time points (Fig. 1f). Taken together, these findings suggest that in the presence of PTX3, ECs attempt to produce energy preferentially through increasing mitochondrial respiratory capacity and by partially sustaining the glycolytic pathway.

## Discussion

The main finding of this study is that PTX3, an acute-phase inflammation protein, induces morphological and bioenergetic changes in isolated, human endothelial cells.

Endothelial cells represent a complex and dynamic system capable of responding to different stimuli, having a wide range of receptors and the ability to produce a series of substances that act at multiple levels. Functions of normal endothelium include the control of vascular tone, thrombosis, and thrombolysis, the production of adhesion molecules, and regulation of the inflammatory response [13]. From this premise, it emerges that ECs constitute a true endocrine–autocrine–paracrine organ. Moreover, being made up of only a monolayer of cells, any alteration of the endothelium has the potential of negatively effecting the cardiovascular system. Thus, considering our previous study demonstrating the deleterious effect that PTX3 has on vascular endothelium, we decide to investigate here its effects on isolated ECs at ultrastructural and metabolic levels.

Among the numerous morphological changes that ECs can undergo, there is extensive literature on the role of vacuolation in the activation of processes such as cell death, lumen formation, and the response to an insult [14, 15]. The most accepted opinion on cytoplasmic vacuolization in ECs is that it represents an adaptive physiological response for damage limitation, in which the cell tries to respond to insults by using any available energy source [16]. Thus, based on this concept and on previous studies showing that PTX3 inhibits EC proliferation and migration – and thus candidating it as a potential anti-angiogenic factor –[17] and that it hampers nitric oxide production [10], the observation here of cytoplasmic vacuolization and concomitant mitochondrial matrix dilution has led us to hypothesize that PTX3 exerts a damaging effect on isolated ECs. In fact, and in agreement with the literature, dilution of the mitochondrial matrix is strictly linked to cellular damage and to an increase in metabolic activity and respiratory state [12].

Data on the role of mitochondrial structural change in ECs is scarce, probably due to the low content of mitochondria in this cell type. However, despite mitochondria composing only 5% of EC volume – contrast this with 28% in hepatocytes – these organelles play an important role in endothelial signalling and function [18]. Indeed, the role of mitochondria goes beyond their capacity to generate the molecular fuel, namely ATP, required for a multitude of cellular processes: they produce reactive oxygen species, regulate calcium activation in cell death, and modulate important endothelial intracellular signalling pathways. Emerging studies suggest that balance in mitochondrial dynamics is relevant to EC structure and function, and its alteration is observed in the endothelium of patients with cardiovascular risk factors [19].

Interestingly, our study of EC bioenergetics revealed that PTX3 induces significant enhancement of cellular metabolism, increasing OxPhos through mitochondrial function and stimulating the glycolytic pathway, processes that are associated with the morphological changes observed and that are typical of the suffering cell in which homeostatic mechanisms to restore the *status quo* has been activated [16].

In conclusion, our study demonstrates the direct effect that PTX3 has on isolated, human endothelial cell homeostasis. The evoked morphological and metabolic changes might represent a compensatory response to cellular damage. This is in agreement with our previous finding of a deleterious effect of PTX3 on the vascular system. More studies are needed to better characterize direct correlation between the ultrastructural and bioenergetic changes induced by PTX3 with endothelial dysfunction, and to better understand the mechanisms involved in age-related cerebro- and cardio-vascular diseases.

## Abbreviations

DMEM: Dulbecco’s Modified Eagle Medium
EBM: Endothelial basal medium
ECAR: Extracellular acidification rate
ECs: Endothelial cells
FCCP: Carbonylcyanide-4-(trifluoromethoxy)phenylhydrazone
HUVECs: Human umbilical vein endothelial cells
OCR: Oxygen consumption rate
OxPhos: Oxidative phosphorylation
PTX3: Pentraxin 3
TEM: Transmission Electron Microscopy
